# The 3D Ultrastructure of *C. elegans* Gut Granules

**DOI:** 10.17912/micropub.biology.002091

**Published:** 2026-04-26

**Authors:** Grant Archer, Sam Bartlowe, Bilegtugs Bayarbadrakh, Dawson Bok, Carolina Bushong, Anna Carroll, Megan Cole, Eric J. Dahl II, Nicholas Denier, Ahmed Elewa, Hafsa El Harchi, Daniel Fisher, Hannah Grant, Aliyah Grijalva, Rachel Grinfeld, Aiden Hale, Cara Hendricks, Melek Iskandar, Sharron Kagan, Hope Keuneke, Lauryn Lammers, Annie Lyons, Quinn Maclin, Nidhi Mistry, Tatum Moore, Connor Munro, Gavin Nickerson, Brando Papalia, Kate Peacock, Lauren Ritzman, Aiden Ross, Rishi Samineni, Max Scales, Lillian Schotz, Wyatt Sikkema, Trinity Slabaugh, Autumn Sorenson, Seyana Sutherland, Raymond Swisher, Daniel Valdes, Faith Ward, Paulina Wojdyla

**Affiliations:** 1 Miami University, Oxford, OH, US; 2 Department of Biology, Miami University, Oxford, OH, US

## Abstract

We identify an endoderm-restricted organelle in published volume electron microscopy datasets of *C. elegans* embryos. The organelle consists of a tubular ring surrounding a membrane-bound compartment harboring a prominent densely stained particle and exhibits a basal polarity concordant with canonical gut granules. This finding offers ultrastructural detail to recent evidence that gut granules are bi-lobed organelles with two distinct compartments.

**Figure 1. A C. elegans endoderm-restricted organelle:** **(a)** Ventral view of annotated organelle (orange) in a bean-stage embryo against a FIB-SEM section (endodermal nuclei, black). The annotated organelles are from the entire depth of the intestine. White box highlights a group of organelles depicted in panel (e). **(b)** A cross-section through the organelle accompanied with a graphical representation clarifying the cross-sections through the tubular ring (blue), the encircled compartment (yellow) and the densely stained particle (magenta). The white dot represents coordinate values x = 889, y = 368 in the bean-stage dataset and the section number is denoted in the top right. **(c)** A cross-section through the organelle neighboring that in (b) but in an orthogonal orientation accompanied with a graphical representation making prominent the tubular ring (blue) encircling the compartment (yellow). The white dot represents the same x, y values noted in (b) and the section number is denoted in the top right. A mitochondrion is labeled, mit. **(d)** Serial sections of the two neighboring orthogonally oriented organelles depicted in (b) and (c). Black arrows and arrowhead indicate tubular ring and densely stained particle, respectively, of the organelle in (b). White arrows and arrowhead indicate tubular ring and densely stained particle, respectively, of the organelle in (c). The section number is noted in the top right corner. **(e)** Four organelles in a bean-stage endodermal cell shown across three sections. These organelles were highlighted with a white box in panel (a). The section number is indicated in the bottom left corner of each crop. White arrow indicates a reconstructed organelle displayed below along three orthogonal axes. Tubular ring (blue), encircled compartment (yellow), densely stained particle (magenta). Vertical and horizontal lines correspond to the noted sections. **(f)** Serial sections of representative organelles in three additional embryonic stages. Each middle section (thick border) consists of two organelles. Arrows denote organelles across sections. The section number is indicated in the top left of each crop. **(g)** Graphical representation of 16 endodermal cells in the bean-stage as observed in the FIB-SEM dataset. Colors distinguish the section from which the cell outline was traced (salmon = 563, turquoise = 467, green = 391, yellow = 307). Apical and basal halves of endodermal cells are clarified using cells Earpa and Ealpa as an example. **(h)** Barplot of asymmetry index of organelle in each of the 16 endodermal cells and the cumulative index combining all 16 indices. Bar positions and colors are matched to the cell positions and colors in (g) to facilitate cross-referencing. **(i)** A model of gut granule ultrastructure. Panels (b – d) are from endodermal cell Earap, and panel (e) is from endodermal cell Eplpp. Scale bars: 10 µm (a); 1 µm (b – f).

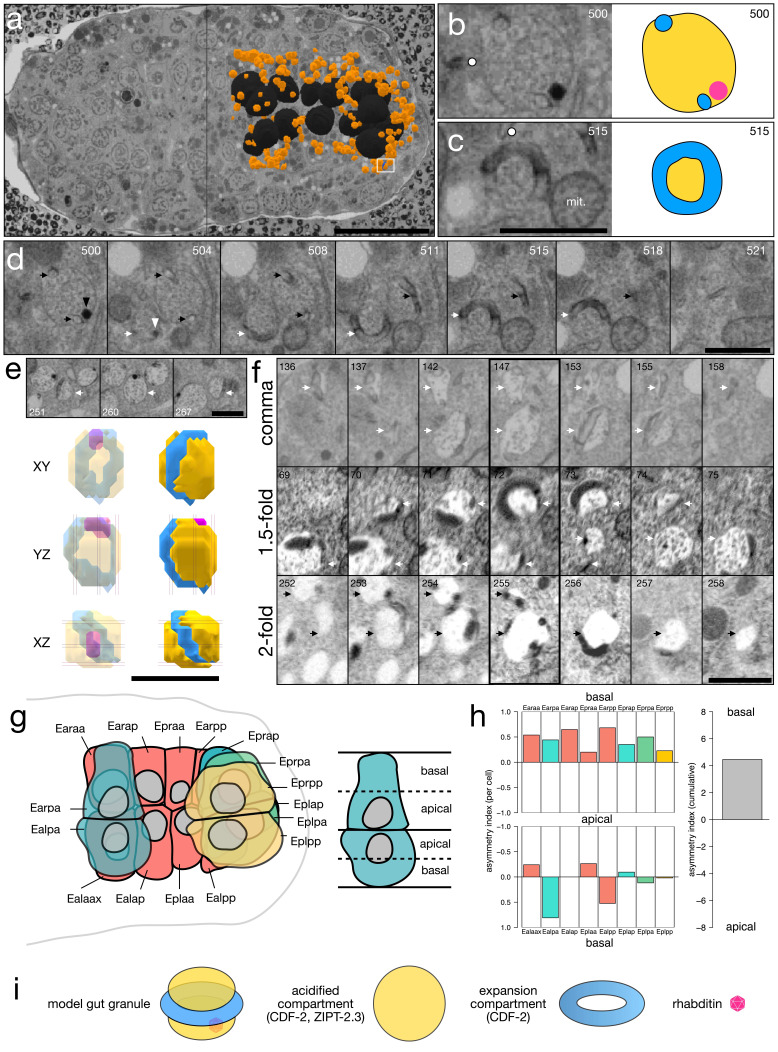



## Description

The entire *C. elegans* intestine is built from endodermal cells that descend from a single blastomere [Sulston et al., 1983]. Restricted to intestinal cells are gut granules, lysosome-related organelles required for trace-metal storage and the synthesis of glycolipid signaling molecules known as ascarosides [Laufer et al., 1980, Hermann et al., 2005, Davis et al., 2009, Panda et al., 2017, Le et al., 2020]. So strict is gut granule confinement to the endoderm lineage that their ectopic appearance is evidence that a cell has aberrantly adopted an endodermal identity [Mello et al., 1992]. Fluorescent metabolites and birefringent crystals have endowed gut granules with striking optical properties [Cobb, 1914, Siddiqui and Babu, 1980]. The tryptophan metabolite anthranilic acid emits blue fluorescence from gut granules, and upon organismal death, floods the cytoplasm of intestinal cells in an anterior-to-posterior wave of morbid blue light [Coburn et al., 2013]. The source of birefringence is rhabditin, a crystallized organic substance originally identified in other nematodes and tentatively reported to fit the chemical profile of carbohydrates but apparently not metabolized upon starvation [Cobb, 1914, Laufer et al., 1980]. Over a century after its initial chemical characterization, the composition of rhabditin crystals remains a mystery. An intriguing property of gut granule structure emerges during zinc homeostasis. During zinc deficiency or excess, gut granules remodel from a predominantly spherical organelle into a bilobed organelle composed of an acidified compartment stained with the lysosome-specific dye LysoTracker and an adjacent expansion compartment [Roh et al., 2012, Mendoza et al., 2024]. Published membrane-marker studies indicate that the Cation Diffusion Facilitator protein CDF-2 is present on both compartments, whereas the zinc ion transporter ZIPT-2.3 is restricted to the acidified compartment and excluded from the expansion compartment, demonstrating that the two lobes are compositionally distinct [Davis et al., 2009, Roh et al., 2012, Mendoza et al., 2024]. Super-resolution microscopy imaging of gut granules suggests that during zinc deficiency or excess, the expansion compartment is often shaped like a hemisphere attached to the acidified compartment. However, whether the expansion compartment fully surrounds the acidified compartment, or whether the latter remains in contact with the cytosol has not been resolved [Mendoza et al., 2024].&nbsp;

During an undergraduate course in cell biology, we sought the three-dimensional ultrastructure of *C. elegans* gut granules by exploring a bean-stage embryo imaged using focused ion beam scanning electron microscopy (FIB-SEM) [Santella et al., 2022]. We identified an organelle that is restricted to endodermal cells and has a composite structure consisting of a tubular ring encircling a membrane-bound compartment harboring a prominent, densely stained particle ( **Figure 1a-e** ). To perform a systematic census of the organelle in question, all organelles that appeared to consist of a round or lenticular compartment encircled by a tubular compartment were annotated ( **Figure 1a** and WEBKNOSSOS link **, Methods, Extended Data** ). The majority (93.3%) of the annotated organelles included the densely stained particle (293/314). We estimate the organelle volume during the bean-stage to be 0.13 ± 0.04 µm ^3^ (mean ± SD) with a typical diameter of 0.63 µm ( **Methods** ). Earlier investigations of *C. elegans* gut granules using confocal microscopy reported a mean diameter of 0.78 µm during the late embryonic pretzel-stage and 1.52 µm in adult gut cells [Barrett and Herman, 2016]. The identified organelle was not observed in any other cell-type in the dataset. Exclusive presence in endodermal cells was also confirmed in a comma-stage embryo imaged with FIB-SEM, and two later stages (1.5-fold and two-fold) imaged with array tomography [Santella et al., 2022] ( **Figure 1f, Methods** ). Importantly, the organelle in question displayed a significant basal enrichment (mean asymmetry index = 0.279, t = 3.339, df = 15, p = 0.004, 95% CI: 0.101, 0.457) consistent with the reported basal polarization of gut granules in the bean-stage [Brandt et al., 2022] ( **Figure 1g-h, Methods** ).

Taken together, we have identified an organelle with a cell-type specificity and asymmetric distribution that corresponds to canonical gut granules. No other observed organelle shared such privilege. The tubular ring invokes the halo-like expansion compartment identified under replete zinc conditions using super-resolution microscopy, whereas the encircled compartment resembles the more compact LysoTracker stained acidified compartment [Mendoza et al., 2024]. The densely stained particle is suggestive of rhabditin. Based on the observed ultrastructure, we propose a model of gut granule biogenesis where a tubular process (perhaps from the endoplasmic reticulum) encircles a low pH lysosome-related compartment to form a dynamic composite organelle ( **Figure 1i** ).

## Methods

Annotation: The volume electron microscopy datasets from [Santella et al., 2022] were made available on WEBKNOSSOS [Boergens et al., 2017]. The links to the datasets retrieved from [Santella et al., 2022] are as follows:

*C. elegans* bean-stage, https://wklink.org/1322

*C. elegans* comma-stage, https://wklink.org/1623

*C. elegans* 1.5-fold stage, https://wklink.org/3489

*C. elegans* two-fold stage, https://wklink.org/7824

The URL to Figure 1a made available on WEBKNOSSOS: https://webknossos.org/links/cS0lvL6wLP597w8P

The operational definition of a presumptive gut granule is a spherical, oval or lenticular membrane-bound compartment that is not a nucleus or mitochondrion and that is encircled by a tubular structure. The presence of the tubular ring eliminates lipid droplets, yolk droplets, endosomes, lysosomes and engulfed corpses from the gut granule category. The densely stained particle is not a defining feature of a presumptive gut granule though it appears in the majority of instances.

Measurement: Since all presumptive gut granules in a given cell were annotated under the same segment, the volume calculated by WEBKNOSSOS represents the volume occupied by all presumptive gut granules in a given cell. Dividing this volume by the number of gut granules in the cell therefore represents the average gut granule volume in a given cell. This average volume ranged from 0.074 µm ^3^ in Ealpp to 0.222 µm ^3^ in Earaa. The mean of averages was 0.130 µm ^3^ and standard deviation 0.037 µm ^3^ . Assuming a spherical gut granule would yield a diameter of 0.629 µm (diameter = 2×(3 *V* /4π) ^1/3^ .

Restriction to endodermal cells: A subset of bean-stage cells were distributed among the authors such that several cells belonging to hypodermis (Caaaap, Caaaaa, ABpraapppp, ABplaappaa, ABplaapppp, Cpaaap, Cpaaaa), muscle (Dpapa, MSappapp, Dppap, MSapppaa, Dpppax, MSappppp, Capaaaa), neuron (ABplaapappp, ABplppaapap, ABplpapapap, ABprppapaaa, ABalpapapap, ABplpaaappp, ABplppaaaaa, ABpraapappp), gut (Earaa, Ealaax, Ealap, Ealpa, Earpp), seam (ABarppppaa, ABarppppap, ABprappapa, ABarpapppp, ABarppaaap) and pharynx (MSaappaa, MSaappap, ABalpappapp, MSaapaaap, ABarapapppa, MSaaappp, ABalpaaappp) cell types in addition to three excretory cells (ABprpaaaapa, ABplpappaap, ABprpapapaa), somatic gonad (Z1/4) and germ cell progenitors (Z2/3) were assigned. Each cell was explored and annotated by the assigned author over the course of the 10 weeks and authors discussed their observations with colleagues assigned a similar cell type as well as colleagues assigned a different cell type. The resulting group conclusion was that the presumptive gut granules were only observed in the gut/endoderm cells. The lead author confirmed the conclusions via a thorough exploration of the bean-stage dataset and annotation of all presumptive gut granules in the 16 endodermal cells. For the comma, 1.5-fold and two-fold stages, the presence of presumptive gut granules in endoderm cells was first confirmed. At least one hour was spent searching for presumptive gut granules in the non-endoderm cells of each embryonic stage and did not encounter any instances.

Basal enrichment: Basal enrichment was determined by calculating an asymmetry index ( *I* ) = ( *N* ^basal^ - *N* ^apical^ )/ ( *N* ^basal^ + *N* ^apical^ ), where *N* = the number of organelles in the basal or apical half of the cell. The basal and apical halves of the cell were determined by displaying the embryo along the XZ axes (i.e., a side view of the embryo rather than ventral view) and then determining the first and last sections of each endodermal cell, representing the span of the cell along the Y axis (i.e., anatomical left/right axis). The apical end of the cell was defined as the end neighboring another endodermal cell, with the opposite end thereby being the basal side. The number of sections was divided by two and the number of organelles annotated in each half was counted. Organelle counting was done twice, the first time proceeding apical to basal and the second in the opposite direction. This measure prevents falsely enriching organelles in the half where counting began and the organelles appeared first. The values from both counts were averaged before calculating the asymmetry index. When the asymmetry index is calculated for each cell individually the value lies between +1 (basal) and -1 (apical). When combined into a cumulative asymmetry index, the value lies between +16 (basal) and -16 (apical). In both cases, an index of 0 means no asymmetry.

Statistical analysis: A one-sample t-test was applied in RStudio (version 2023.06.1+524) to determine whether the mean asymmetry index significantly differs from zero.

## Data Availability

Description: Annotation data downloaded from WEBKNOSSOS including volume annotations as Zarr.. Resource Type: Dataset. DOI: https://doi.org/10.22002/9m482-9br85
